# A Taiwanese Nationwide Cohort Study Shows Interferon-Based Therapy for Chronic Hepatitis C Reduces the Risk of Chronic Kidney Disease

**DOI:** 10.1097/MD.0000000000001334

**Published:** 2015-08-14

**Authors:** Yi-Chun Chen, Shang-Jyh Hwang, Chung-Yi Li, Chia-Pin Wu, Li-Chu Lin

**Affiliations:** From the Division of Nephrology, Department of Internal Medicine, Dalin Tzu Chi Hospital, Buddhist Tzu Chi Medical Foundation, Chiayi (Y-CC, L-CL); School of Medicine, Tzu Chi University, Hualien (Y-CC); Division of Nephrology, Department of Internal Medicine, Kaohsiung Medical University Hospital, Kaohsiung (S-JH); Department and Graduate Institute of Public Health, College of Medicine, National Cheng Hung University, Tainan (C-YL); Department of Public Health, College of Public Health, China Medical University, Taichung (C-YL); and Public Health Department, New Taipei City Government, Taipei, Taiwan (C-PW).

## Abstract

Hepatitis C virus (HCV) infection is a risk factor for chronic kidney disease (CKD). However, it remains unclear whether interferon-based therapy (IBT) for HCV was associated with reduced risk of CKD.

From the Taiwan National Health Insurance Research Database, we identified 919 patients who received 3 months or more of IBT as our treated cohort. This cohort was propensity score-matched 1:4 with 3676 controls who had never received IBT for HCV infection (untreated cohort). Cumulative incidences of and hazard ratios (HRs) for CKD were calculated after adjusting for competing mortality.

In the matched HCV cohort, the risk of CKD was significantly lower in the treated cohort (7-year cumulative incidence, 2.6%; 95% confidence interval [CI], 0.7%–6.9%) than in the untreated cohort (4%; 95% CI, 3.5%–5.2%) (*P* < 0.001), with an adjusted HR of 0.42 (95% CI, 0.20–0.92; *P* = 0.03). The results also held in the overall HCV cohort. The number needed to treat for 1 fewer CKD at 7 years was 58. The reduced risk of CKD was greatest (0.35; 0.14–0.87; *P* = 0.024) in HCV-infected patients who received 6 months or more of IBT. Multivariable stratified analysis verified that greater risk reduction of CKD was present in HCV-infected patients with hyperlipidemia, diabetes, hypertension, and those without coronary heart disease.

In conclusion, IBT, especially for 6 or more months, is associated with reduced risk of CKD in HCV-infected patients. Hyperlipidemia, diabetes, hypertension, and coronary heart disease can modify this association.

## Abstract 2

Supplemental Digital Content is available in the text

## INTRODUCTION

Hepatitis C virus (HCV) infection and chronic kidney disease (CKD) are major public health issues in Taiwan and worldwide.^1,2^ Apart from major liver complications, mounting evidence indicates that HCV adversely affects renal function. HCV is associated with increased risks of CKD^3^ and end-stage renal disease (ESRD),^4^ even in the absence of cirrhosis.^3^ HCV also accelerates progression of CKD to ESRD in patients with glomerulonephritis or diabetes.^5,6^

Studies have reported that HCV infection can induce a state of oxidative stress and overproduces proinflammatory cytokines that play a critical role in insulin resistance.^7^ Compensatory hyperinsulinemia in an insulin-resistant state further enhances oxidative stress and promotes endothelial dysfunction, which contributes to renal injury.^8^ These observations suggest a biologically plausible mechanism for increased risk of CKD in certain HCV-infected subjects. Up to 70% of HCV-infected patients with and without cirrhosis display insulin resistance,^9,10^ which is predominately extrahepatic.^11^ Interferon-based therapy (IBT) is considered the mainstay of HCV treatment.^12^ HCV eradication ameliorates insulin resistance^13,14^ and oxidative stress.^15^ Therefore, it seems plausible that HCV-infected patients receiving IBT also have decreased risk for CKD.

To date, there have been no nationwide cohort studies regarding the impact of IBT on CKD risk in HCV-infected patients, and it is unclear if certain subsets of HCV-infected patients receiving IBT are more likely to have decreased CKD risk. Taiwan is a particularly suitable setting for examining the relationship of IBT for HCV with CKD because it has a high prevalence of both conditions.^1,2^ Moreover, the burden of CKD and HCV infection is rising annually.^1^ We hypothesized that IBT for HCV would reduce CKD risk, given the efficacy of IBT in ameliorating insulin resistance and oxidative stress.^13,15^ Hence, we examined this association using reimbursement claims data from the Taiwan National Health Insurance Research Database (NHIRD) during a follow-up period of 7 years.

## METHODS

### Database

This cohort study used outpatient and inpatient claims from the NHIRD from 1996 to 2010, which is released by the National Health Research Institutes for Taiwan's National Health Insurance (NHI) Program. The NHI is a government, compulsory-enrolment, single-payer system that had a coverage rate of more than 99% by the end of 2010, and adopts ICD-9 diagnosis codes for provider payment applications. The NHIRD lacks information on laboratory and lifestyle data and severity of the disease condition. Our previous research provided details of the NHIRD.^3,16–19^ In brief, the NHIRD has detailed healthcare data of 25.68 million enrollees (99.9% of the population of Taiwan) based on a random sample of all enrollees of the NHI program. There were no significant differences in age, sex, or healthcare costs between the sample group and all enrollees. The NHI Administration performs a medical quality monitoring and assurance program every month, including chart reviews, charge audits, and heavy penalties for inappropriate charges or malpractice. Therefore, it is generally believed that these checks and balances can ensure accurate coding and further minimize misclassification error.^18,20^ The study was approved by our institutional review board. Informed consent was not required because this is a secondary data analysis.

### Study Population

We identified all patients who had a first-time diagnosis of HCV infection (ICD-9-CM codes 070.41, 070.44, 070.51, 070.54, V02.62)^3,21^ between January 1, 2004 and December 31, 2007 from the outpatient and inpatient claims. A total of 9639 HCV-infected patients were identified. Patients, who were aged less than 18, had claim-based diagnoses of HCV before January 1, 2004, of HBV (ICD-9-CM codes 070.22, 070.23, 070.32, 070.33, V02.61)^18^ and renal transplantation (ICD-9-CM code V420) from 1996 to 2010, or of CKD and IBT received before the first HCV diagnosis were excluded, resulting in a total of 8810 patients with newly diagnosed HCV.

### Study Cohorts

The HCV cohort was divided into 2 cohorts based on the use of IBT, including interferon alpha, pegylated interferon alpha-2a, and pegylated interferon alpha-2b.^21^ The NHI Administration has been reimbursing IBT (Table S1, http://links.lww.com/MD/A375) for HCV for 6 months’ duration for all genotypes since October 1, 2003.^21–23^ Thus, patients who received IBT for 3 months or more^21^ were designated as the treated cohort (n = 919). As the combination of ribavirin and interferon is a common treatment regimen, we also extracted the use of ribavirin; most patients (98.9%) were prescribed combination therapy. The index date of the treated cohort was defined as the date of the start of IBT. Patients who never received IBT between 2004 and 2010 were designated as the untreated cohort (n = 7828). The index date of the untreated cohort was defined as the first occurrence of an HCV claim during the entry period. Thus, the overall HCV cohort included 8747 patients.

For each treated patient, 4 untreated patients were selected at or after the day when IBT was initiated in the treated cohort according to the propensity score that was calculated to adjust for the baseline differences between patients with and those without IBT. The propensity score was estimated by the logistic regression built on the baseline variables including age, sex, comorbidities, geographic region, urbanization level, enrollee category (EC), number of medical visits, and Deyo–Charlson comorbidity index (CCI) score. The propensity score model was reliable (Hosmer–Lemeshow test *P* = 0.06) and provided fair discrimination between the cohorts (c-index, 0.63).^18,24^ Thus, the matched HCV cohort included 4595 patients.

### Definition of CKD

The claims-based diagnosis of CKD was defined by the presence of 1 inpatient or 2 outpatient ICD-9 code 585^3,25^ in the claims and without catastrophic illness registration cards for ESRD (indicating the need for renal replacement therapy). The ICD-9 code 585 is consistent with the Kidney Disease Outcomes Quality Initiative/Kidney Disease: Improving Global Outcomes definition of CKD stages 1–5, which allows for comparisons of the incidence and prevalence of CKD in Taiwan and the United States.^3,25^ However, the CKD stage (severity) cannot be assessed in the NHIRD.

### Main Outcome Measurement

Both cohorts were followed from the index date to the first diagnosis of CKD, death, or the end of 2010, whichever came first. Because IBT has been shown to decrease mortality in HCV-infected patients,^26^ censoring resulting from death was regarded as informative and was adjusted by using competing risk analyses. Death was defined by withdrawal from the NHI program.^18,27^

### Potential Confounders

We recorded the claims-based diagnoses of comorbidities associated with CKD between January 1, 1996 and the index date according to ICD-9 codes, including diabetes (ICD-9 code 250), hypertension (ICD-9 codes 401-405), coronary heart disease (ICD-9 codes 410-414), hyperlipidemia (ICD-9 codes 272-272.4), and cirrhosis (ICD-9 codes 571.2, 571.5, 571.6).^3^ CKD was associated with geographic region of residence and socioeconomic status.^3^ Thus, we recorded geographic regions (northern, central, southern, or eastern Taiwan) in order to reduce potential confounding by differential accessibility of medical care,^3,16,18^ and urbanization level (urban, suburban, and rural) and EC, from EC1 (highest status) to EC4 (lowest status), as proxy measures of socioeconomic status, to minimize environmental effects.^3,18^ We also considered the number of medical visits^3,16,18,27^ as a potential confounder and used the CCI score for control of confounding in studies using administrative databases.^18,28^ Finally, we considered propensity score in regression adjustment to control for confounding in healthcare utilization databases^18,29^ and to reduce bias in the background covariates between the 2 cohorts.^18,30^

### Statistical Analyses

We calculated and compared the cumulative incidences of CKD by use of the modified Kaplan–Meier method and Gray method,^31^ and tested differences in the full time-to-event distributions between the study cohorts using log-rank test. The number needed to treat (NNT) represented the number of patients needed to be treated to yield 1 fewer CKD; the NNT was calculated with the inverse of the absolute risk reduction.^32^ After ensuring the assumption of proportional hazards, we used the modified Cox proportional hazard model to examine the association of IBT with CKD risk,^33^ with adjustment for all covariates (age per year, sex, comorbidities, geographic region, urbanization level, EC, number of medical visits, CCI score, and propensity score). We further performed a stratified analysis of the effect of IBT on CKD risk and compared the effect of IBT duration (<6 vs ≧6 months) on CKD risk in the propensity score-matched HCV cohort. We analyzed all data with SAS (version 9.2; SAS Institute, Inc., Cary, NC) and considered a 2-sided *P*-value less than 0.05 as statistically significant.

## RESULTS

### Baseline Characteristics of the HCV Cohort

Baseline characteristics and follow-up status of the overall and matched HCV cohorts are presented in Table 1. The mean (±SD) interval from HCV diagnosis to start of IBT was 2.4 ± 2.0 years, and the mean duration of IBT was 0.6 ± 0.9 years. In the matched HCV cohort, there were no significant differences of baseline covariates between the 2 cohorts, except for EC; the treated cohort had a higher percentage of EC3. In the overall or matched HCV cohort, the percentage of CKD events and competing mortality was lower in the treated cohort than in the untreated cohort (all *P* < 0.0001).

**TABLE 1:**
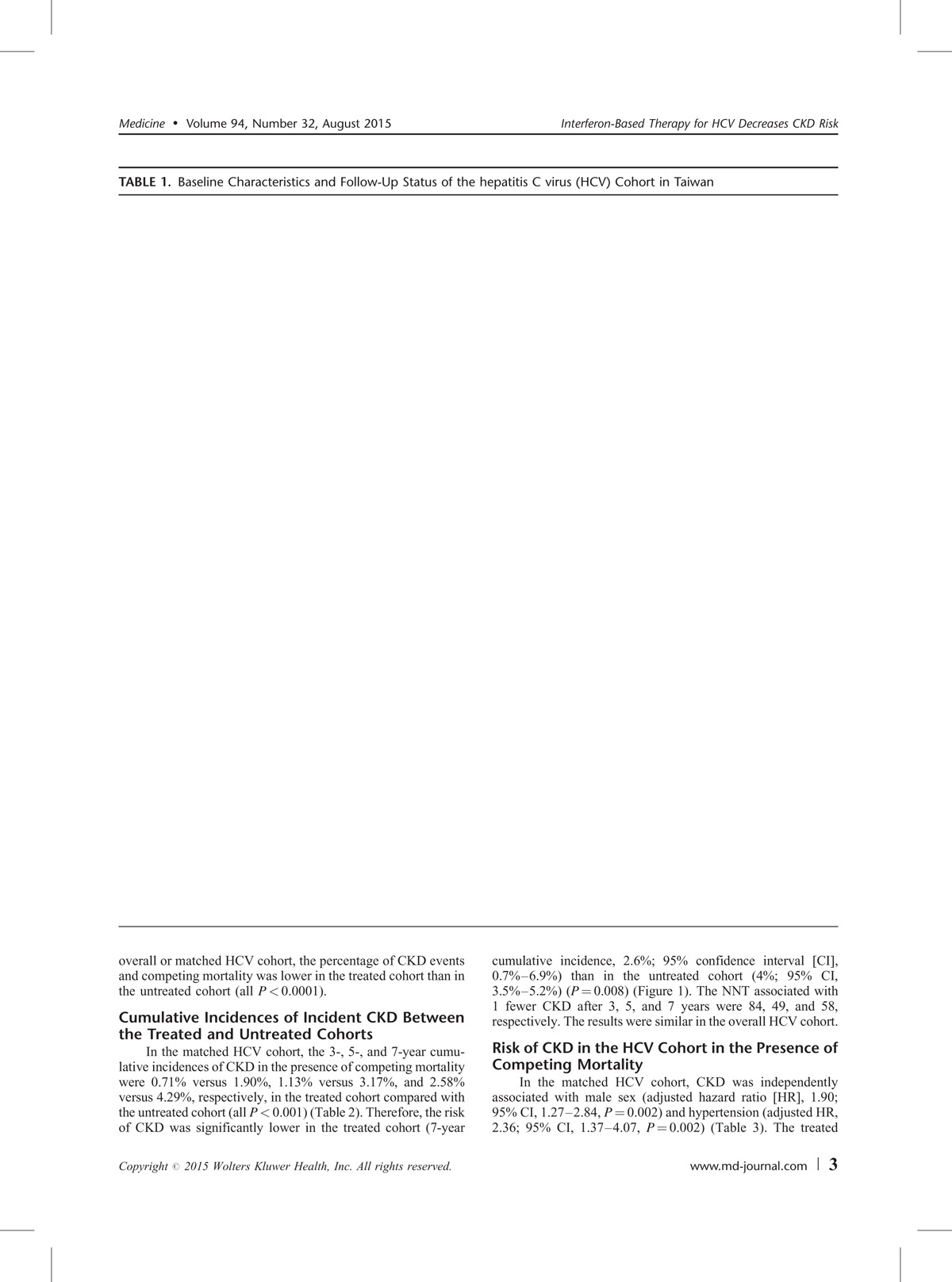
Baseline Characteristics and Follow-Up Status of the hepatitis C virus (HCV) Cohort in Taiwan

### Cumulative Incidences of Incident CKD Between the Treated and Untreated Cohorts

**FIGURE 1:**
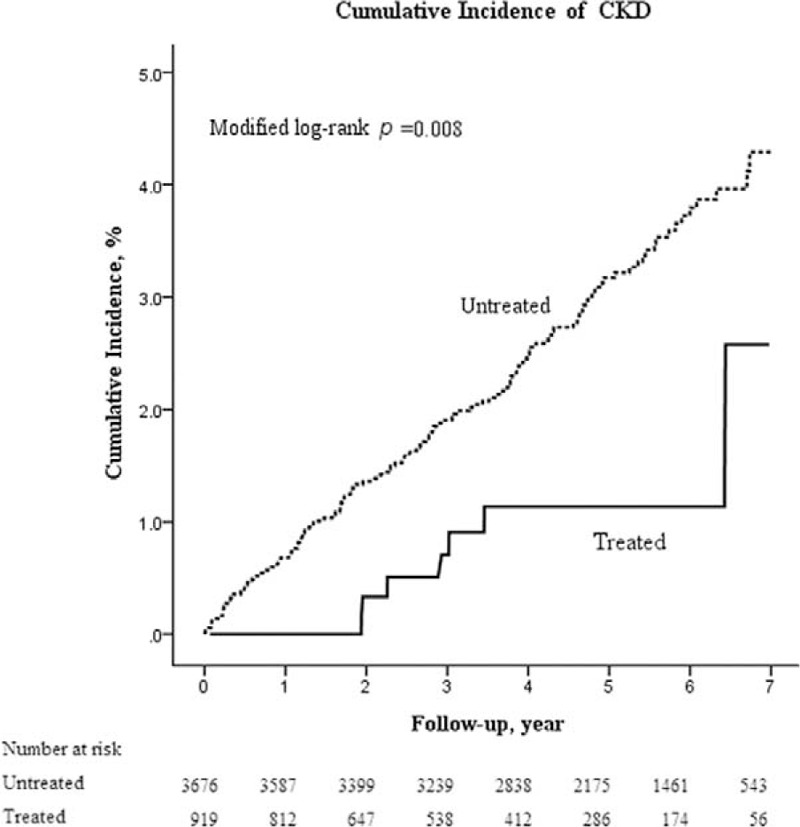
Cumulative incidence of CKD in the propensity score-matched HCV cohort with (treated, solid line) and without (untreated, dash line) interferon-based therapy. Data were compiled after adjustment for competing mortality. CKD = chronic kidney disease, HCV = hepatitis C virus.

In the matched HCV cohort, the 3-, 5-, and 7-year cumulative incidences of CKD in the presence of competing mortality were 0.71% versus 1.90%, 1.13% versus 3.17%, and 2.58% versus 4.29%, respectively, in the treated cohort compared with the untreated cohort (all *P* < 0.001) (Table 2). Therefore, the risk of CKD was significantly lower in the treated cohort (7-year cumulative incidence, 2.6%; 95% confidence interval [CI], 0.7%–6.9%) than in the untreated cohort (4%; 95% CI, 3.5%–5.2%) (*P* = 0.008) (Figure 1). The NNT associated with 1 fewer CKD after 3, 5, and 7 years were 84, 49, and 58, respectively. The results were similar in the overall HCV cohort.

**TABLE 2:**
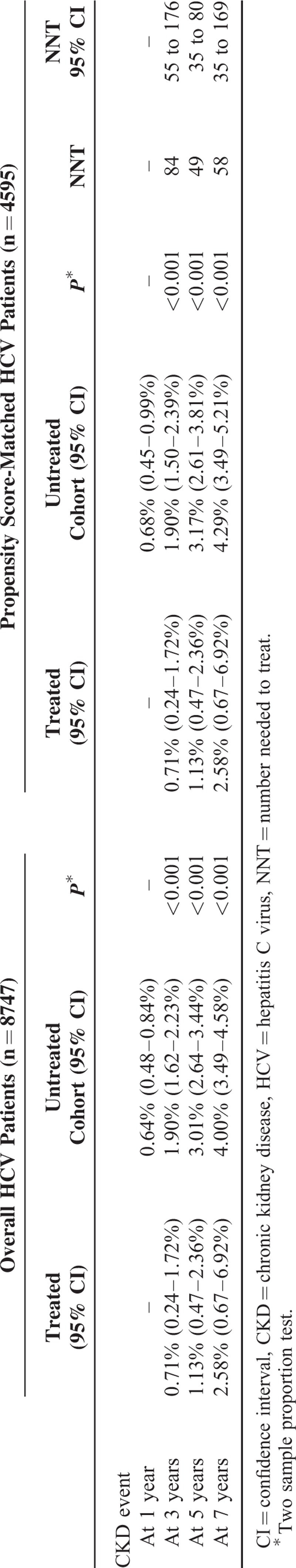
Cumulative Incidences of CKD in the HCV Cohort With and Without Interferon-Based Therapy

### Risk of CKD in the HCV Cohort in the Presence of Competing Mortality

In the matched HCV cohort, CKD was independently associated with male sex (adjusted hazard ratio [HR], 1.90; 95% CI, 1.27–2.84, *P* = 0.002) and hypertension (adjusted HR, 2.36; 95% CI, 1.37–4.07, *P* = 0.002) (Table 3). The treated cohort had a significantly lower risk of CKD than the untreated cohort (adjusted HR, 0.42; 95% CI, 0.20–0.92, *P* = 0.03). The results were similar in the overall HCV cohort. We further performed a sensitivity analysis to test the robustness of our result. We analyzed the treated cohort receiving IBT for 6 months or more and the untreated cohort in the overall and matched HCV cohort and also obtained a similar and significant result (data not shown).

**TABLE 3:**
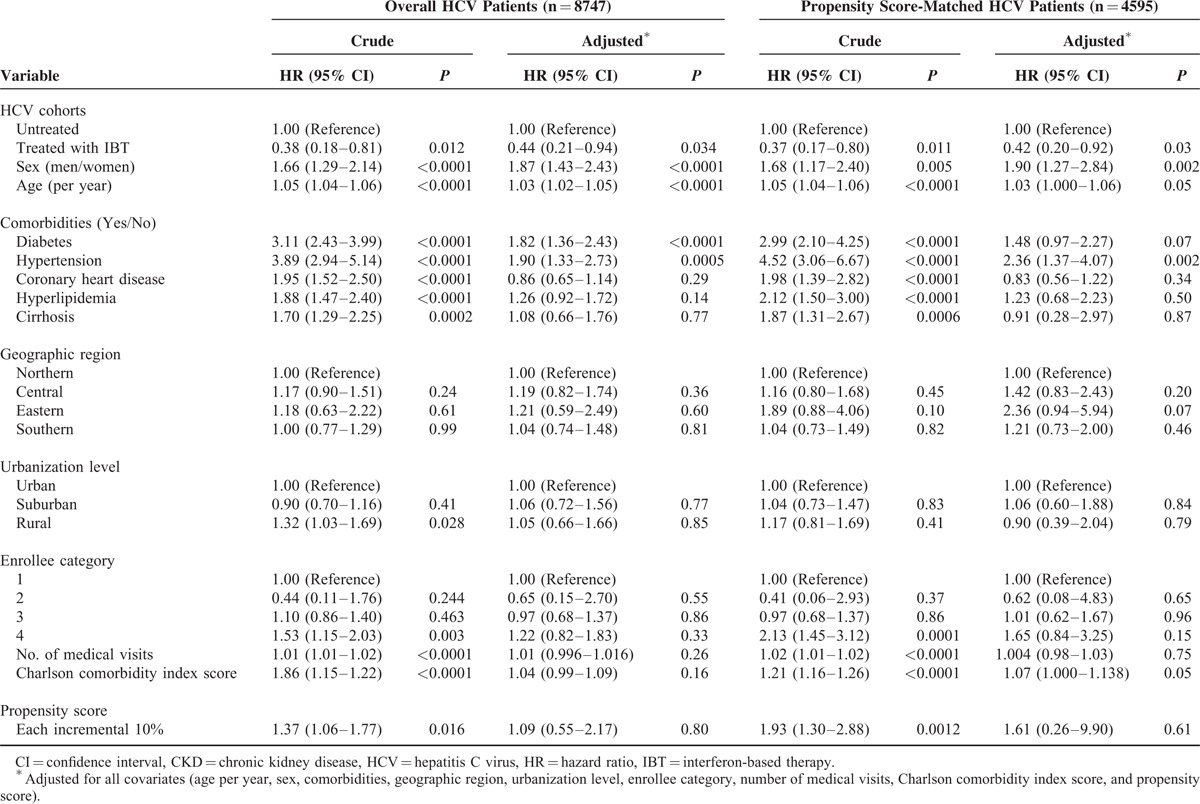
Crude and adjusted HRs for CKD in the HCV Cohort, With Adjustment for Competing Mortality

### Impact of IBT Duration on the Risk of CKD

In the matched HCV cohort, the treated cohort who received 6 months or more of IBT had a significantly lower risk of CKD (adjusted HR, 0.35; 95% CI, 0.14–0.87; *P* = 0.024) (Table 4), compared with the untreated cohort and the treated cohort who received less than 6 months of IBT.

**TABLE 4:**
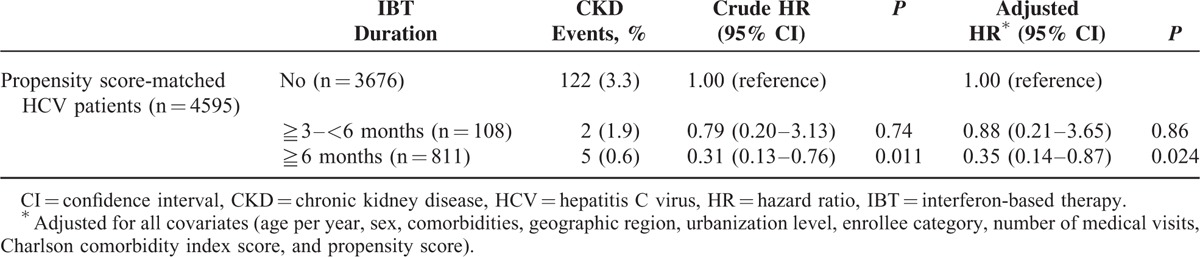
The Effect of Duration of IBT for HCV Infection on Risk of CKD, With Adjustment for Competing Mortality

### Stratified Analysis

**FIGURE 2:**
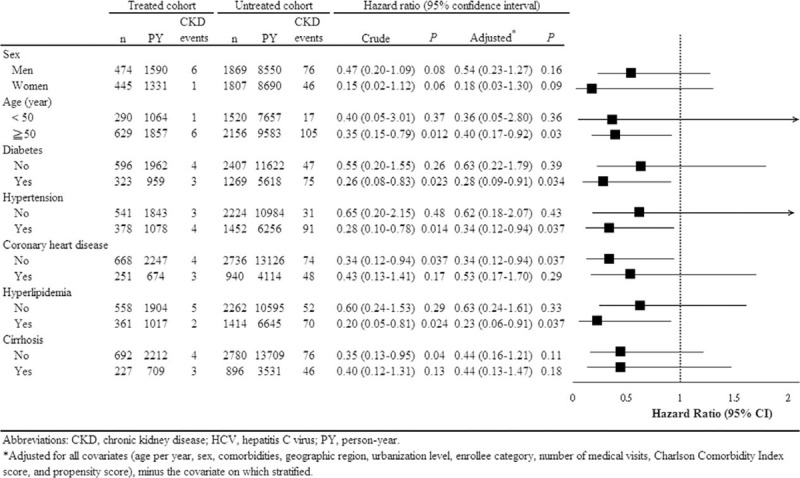
Stratified analysis for the risk of CKD in association with interferon-based therapy in the propensity score-matched HCV cohort, with adjustment for competing mortality. CKD = chronic kidney disease, HCV = hepatitis C virus.

In the matched HCV cohort, IBT was consistently associated with decreased risk of CKD across all subgroups (Figure 2). The reduction of risk was significant in subjects with (adjusted HR, 0.23; 95% CI, 0.06–0.91; *P* = 0.037) versus without (adjusted HR, 0.63; 95% CI, 0.24–1.61; *P* = 0.33) hyperlipidemia, with (adjusted HR, 0.28; 95% CI, 0.09–0.91; *P* = 0.034) versus without (adjusted HR, 0.63; 95% CI, 0.22–1.79; *P* = 0.39) diabetes, with (adjusted HR, 0.34; 95% CI, 0.12–0.94; *P* = 0.037) versus without (adjusted HR, 0.62; 95% CI, 0.22–1.79; *P* = 0.39) hypertension, without (adjusted HR, 0.34; 95% CI, 0.12–0.94; *P* = 0.037) versus with (adjusted HR, 0.62; 95% CI, 0.18–2.07; *P* = 0.43) coronary heart disease, and subjects older (adjusted HR, 0.40; 95% CI, 0.17–0.92; *P* = 0.03) versus younger (adjusted HR, 0.36; 95% CI, 0.05–2.80; *P* = 0.36) than 50 years. However, the magnitude of risk reduction was more pronounced in the first 4 comorbidities.

## DISCUSSION

The most important finding of the nationwide cohort study is not only to demonstrate that IBT for HCV infection was significantly associated with a 58% reduction of incident CKD risk over a 7-year study period after propensity score matching and adjustment for potential confounders and competing mortality; and that longer treatment duration (at least 6 months) may be required for IBT to exert its protecting effect on CKD, but also to characterize HCV-infected patients who are more likely to benefit from this treatment. The cumulative incidence of CKD was significantly lower in HCV-infected patients receiving IBT than in those without IBT. The NNT in association with 1 patient free of CKD at 3, 5, 7 years after IBT was 84, 49, and 58, respectively. The attenuated risk of CKD was more pronounced in HCV-infected patients with diabetes, hypertension, hyperlipidemia, and those without coronary heart disease. These findings suggest that HCV infection may have a role in the pathogenesis of renal injury, and also implicate that treatment of HCV infection may improve renal outcome. This information has important clinical implications for the design of surveillance programs that assess HCV infection and CKD and for the development of clinical practice guidelines.

Reported studies^23,34,35^ that address the association of IBT for HCV with renal outcome are few and lacking detail. A hospital-based retrospective cohort study^34^ analyzed 650 HCV-infected cirrhotic Japanese patients who received IBT for periods of 4–52 weeks and had normal renal function 3 months after IBT termination; the authors further divided them into sustained virological response (SVR) and non-SVR groups. The authors found that the development of CKD was associated with non-SVR rather than with HCV genotype, ribavirin combination, and type of interferon during a mean follow-up period of 6.5 years. However, IBT intervention for protecting new development of CKD was not evaluated in that study. A hospital-based cross-sectional study^35^ analyzed 552 HCV-infected American patients; the authors found that 2.5% of 159 HCV-infected patients who had ever received IBT and 12.5% of 393 HCV-infected patients without IBT developed CKD during a 7-year follow-up period, and indicated that history of IBT was associated with reduced risk of CKD (odds ratio, 0.18; 95% CI, 0.06–0.56). However, the authors did not report the kind and duration of IBT and the effect of IBT intervention and SVR on CKD risk. A nationwide Taiwanese cohort study^23^ indicated that IBT for HCV used for at least 4 months was associated with reduced risk of ESRD (HR, 0.16; 95% CI, 0.07–0.33) in a diabetic cohort without significant comorbidities during an 8-year follow-up period. However, this result may not be extrapolated to most HCV-infected patients because the HCV population is highly comorbid.^36^ Moreover, the authors did not provide NNT associated with 1 patient free of ESRD. We believe that our results can be generalized to HCV population because we did not exclude the HCV cohort with significant comorbidities; the method used to find our HCV cohort was similar to that of a prior NHIRD-based nationwide study of HCV cohort.^21^ Moreover, to evaluate the effect of IBT intervention for HCV on CKD risk, we used a large nationwide dataset, which afforded considerable statistical power and allowed long-term tracking of incident CKD events. We evaluated the number of patients needed to be treated with IBT for 3 months or more for 1 additional patient to benefit, which was not evaluated in any of the 3 above-mentioned studies.^23,34,35^ Although the NNT for 1 fewer CKD at 7 years was 58 in our study, the overall reduction in CKD burden from the HCV population may be substantial, given that 3 to 4 million people are newly infected each year^37^ and the incidence of CKD was 1.66-fold higher in an HCV cohort than a non-HCV cohort.^3^

The exact mechanism that IBT for HCV is associated with reduced CKD risk is unclear. However, the effect of IBT for HCV on the amelioration of insulin resistance may underlie the association revealed in this study. Most HCV-infected patients with and without cirrhosis have insulin resistance and compensatory hyperinsulinemia,^10^ which is associated with increased oxidative stress and endothelial dysfunction, and subsequent renal injury.^8^ The mechanism through which IBT alleviates insulin resistance is most likely mediated via viral eradication,^23^ and SVR is an indicator of successful HCV eradication and clinical cure.^14^ Mounting evidence suggests that attainment of SVR decreases insulin resistance^13,14^ that occurs predominantly in extrahepatic sites^11^ and oxidative stress markers^15^ in HCV-infected patients. Further research is warranted to better understand the mechanism because this study could not directly examine the status of insulin resistance.

Insulin resistance is a hallmark of hyperlipidemia, diabetes, and hypertension, all of which are components of the metabolic syndrome.^38^ This relationship may account for our results that HCV-infected patients who had hyperlipidemia, diabetes, and hypertension obtained more benefits of IBT in CKD risk. The difference in sex had no influence on achieving SVR,^39^ which may account for our result that there was no beneficial difference of IBT on CKD risk in male and female HCV-infected patients. We also found that there was no beneficial difference of IBT on CKD risk in HCV-infected patients with and without cirrhosis, a result that is inconsistent with those of 1 previous study.^23^ Further research is warranted to better understand this similarity in outcome between cirrhotic and noncirrhotic patients. Even though the NHIRD lacks individual information on HCV genotype and SVR,^3,23^ and we could not directly show how SVR influences the above-mentioned associations, we believe that the lower CKD risk resulted from HCV elimination in the treated cohort. Thus, we are confident of IBT's efficacy in the treated cohort, because IBT generally achieves SVR exceeding 70% in Taiwan, where a favorable genetic variation in interleukin-28B is prevalent.^23^ Moreover, there have been several NHIRD-based nationwide cohort Taiwanese studies indicating the benefit of IBT on HCV-related liver and extrahepatic complications.^21,23,26^

The major strength of our study is that it was designed to reduce selection bias (through the use of a large nationwide and highly representative sample with random sampling and the use of propensity score matching to optimize comparability); reduce environmental effects (because of the availability of socioeconomic indicators for all subjects);^3,16,18^ avoid detection bias (because of the universal availability of medical services);^3,18,27^ avoid immortal time bias^40^ (because the time when patients received IBT was chosen as the entry of observation); and prevent overestimation of nonfatal outcomes in the untreated cohort by using competing risk analysis.^18,23^ In addition, the study population was well defined and follow-up was complete because our design relied on the universal coverage of Taiwan's NHI, which fully reimbursed IBT for HCV treatment and thus minimized disparity in healthcare accessibility or financial status as a determinant for receiving IBT. Although unmeasured confounders may still exist, as with any observational study, we believe the method we used are solid and our finding of decreased risk of CKD following IBT for HCV-infected subjects is valid.

Our study had some limitations. First, we were unable to document the adverse reactions related to IBT. Nevertheless, we enrolled HCV-infected patients receiving 3 months or more of IBT^21^ into our analysis to exclude most noncompliant patients. Second, the actual compliance with medication was unknown. Nonetheless, excessive prescription is impossible because of the strict regulations for IBT in Taiwan. Third, misclassification of diseases may occur when an administration database is used. However, the NHI Administration established an audit and penalty system for quality monitoring and assurance to ensure accuracy of claims.^20^ Moreover, both CKD and viral hepatitis are important health problems in Taiwan, so the government has strict guidelines for diagnosis,^41^ and the diagnoses of CKD and HCV by ICD-9 codes have been applied in several NHIRD-based nationwide cohort studies.^3,23,26^ We also adopted the standard methodology (1 inpatient or 2 outpatient diagnosis codes) to capture CKD patients in claims data.^25^ Fourth, the NHIRD lacks information on family history of kidney diseases, lifestyle, body weight, and laboratory data (eg, SVR, HCV RNA and genotype, urinalysis, and serum creatinine, alanine transaminase, aspartate transaminase, albumin, and bilirubin). Thus, we could not include these variables in the PS analysis and clarify the relationships of SVR, obesity, CKD severity (stage), viral count, and genotype with CKD risk. Nevertheless, we added CCI score into the propensity analysis and included CCI score and PS in the regression analysis to control confounding in healthcare administrative databases.^18,27,28,42,43^ This method had been used in previous NHIRD-based research on patients with chronic hepatitis B or C.^18,27,44^ Moreover, we used propensity score matching to minimize allocation bias in order to reach the comparability of the treated and untreated cohorts,^45^ because propensity score, defined as the conditional probability of being treated given the measured covariates, can be used to balance the covariates in the treated and untreated groups,^45^ and propensity matching is an effective method of pseudo-randomization in the treated and untreated groups when the effects of treatment and interventions are compared.^28^ This method had been used in previous nonrandomized observational studies based on healthcare administrative databases^18,23,24,27^ for the same purpose. Finally, although the SVR rates to IBT between the Asian and Western non-HCV genotype-1 (HCV-1) patients are comparable, the SVR rate to IBT in Asian HCV-1 patients is higher than that in Western HCV-1 patients,^37^ largely as a result of interleukin-28B genotypic polymorphism.^23^ Thus, caution is recommended before applying our results to the West.

In conclusion, this national cohort study indicates that CKD risk reduction is greater in HCV-infected patients who receive IBT, as compared with those who do not receive IBT, especially in HCV-infected patients receiving IBT for 6 months or more and in those with hyperlipidemia, diabetes, hypertension, and without coronary heart disease. These findings may offer clinical suggestions to justify the long-term use and renal benefit of IBT in HCV-infected patients and also imply that HCV infection may have a pathogenic role in the development of CKD. Further research is warranted to better understand the causal relationship and pathological mechanism underlying this association.
